# The Combined Use of Stem Cells and Platelet Lysate Plasma for the Treatment of Erectile Dysfunction: A Pilot Study–6 Months Results

**DOI:** 10.3390/medicines7030014

**Published:** 2020-03-18

**Authors:** Vassilis Protogerou, Sara El Beshari, Efstathios Michalopoulos, Panagiotis Mallis, Dimosthenis Chrysikos, Alexandros A. Samolis, Catherine Stavropoulos-Giokas, Theodoros Troupis

**Affiliations:** 1 Department of Anatomy, School of Medicine, National and Kapodistrian University of Athens, M. Asias 21 st, 12462 Athens, Greece; dixrys@yahoo.gr (D.C.); alexsamolis@me.com (A.A.S.); ttroupis@gmail.com (T.T.); 2 2nd Urological Department, Attikon Hospital, Medical School of Athens, National and Kapodistrian University, 12462 Athens, Greece; 3 Health Plus Genomics Laboratory, Part of Health Plus Network of Specialty Centers, 11th St, Hazaa bin Zayed St, Al Karama Area - Abu Dhabi, UAE; sarahbishari@hotmail.com; 4 Hellenic Cord Blood Bank, Biomedical Research Foundation Academy of Athens, 4 Soranou Ephessiou Street, 11527 Athens, Greece; smichal@bioacademy.gr (E.M.); pmallis@bioacademy.gr (P.M.); cstavrop@bioacademy.gr (C.S.-G.)

**Keywords:** erectile dysfunction, stem cells, platelet lysate plasma, adipose-derived stem cells

## Abstract

**Background:** The current treatment of Erectile Dysfunction (ED) is mainly based on the use of drugs that provide erections shortly after use but they do not really treat the problem. Stem cell therapy is a novel treatment with regenerative properties that can possibly treat erectile dysfunction. **Methods:** Five patients with erectile disease were treated with Adipose-Derived Stem Cells (ADSCs) and Platelet Lysate Plasma (PLP). ADSCs were obtained through abdominal liposuction and PLP was prepared after obtaining blood samples from peripheral veins. Erectile function was evaluated with the International Index of Erectile Function questionnaire (IIEF-5) questionnaire, penile triplex at the 1st, 3rd, 6th and 12th month post-treatment. A CT scan of the head, thorax and abdomen was done before treatment and at the 12th month. **Results:** IIEF-5 scores were improved in all patients at the 6th month although not in the same pattern in all patients. Peak Systolic Velocity (PSV) also improved at the 6th month in all patients but also with different patterns in each patient, while End Diastolic Velocity (EDV) was more variable. Two patients decreased the treatment they used in order to obtain erection (from Intracavernosal injections (ICI) they used PDE-5Is), two had unassisted erections and one had an initial improvement which decreased at the 6th month. There were no side effects noted. **Conclusions:** Stem cell therapy in combination with PLP appears to show some improvement in erectile function and has minimal side effects in the short term.

## 1. Introduction

Erectile dysfunction (ED) prevalence was found to be 52% in the Massachusetts Male Aging Study (MMAS) while in another study in the new onset cases, one in four patients was below the age of 40 [1]. The most common risk factors for ED are obesity, diabetes mellitus, dyslipidaemia, metabolic syndrome, lack of exercise, smoking, and it is common in cardiovascular disease [1]. Vascular ED is mainly due to endothelial dysfunction and the currently available treatments, although they can improve erectile dysfunction, cannot be considered as curative. Stem cell therapy is a novel treatment aiming at restoring endothelial dysfunction and thus, provides a possible cure for ED.

The corpora cavernosa play a significant role in establishing an erection [2]. Corpora cavernosa consist of sinusoids, which are covered by a single layer of endothelial cells (ECs), multiple layers of cavernous smooth muscle cells (CSMCs), and the cavernous nerves (CNs) [2,3]. Once stimulated, the activated neuronal NOS (nNOS) produces NO that leads to the relaxation of the cavernosal smooth muscle cells (CSMCs) and as a consequence blood fills up the sinusoids [2,4]. Furthermore, ECs are expressing significant levels of NO during intercourse, which maintain CSMCs in this induced state [2,5]. Damage in key components of erections, such as endothelial cells (ECs), cavernous smooth muscle cells (CSMCs) and neuronal cells can lead to ED [2,6,7,8].

Until now, ED treatment included mainly the use of various pharmaceutical agents with the most widely used being phosphodiesterase type-5 inhibitors (PDE5-I) [5].

The use of the pharmaceutical agents cannot be considered as curative, since the tablets must be taken before sexual intercourse in order to have an erection. These tablets, if taken regularly, can improve the erectile function but even in this case they do not treat the disease, they simply improve endothelial function. This also includes additional cost and is far from curative. Thus, treatment must be found than can actually treat ED. For this purpose, mesenchymal stromal cells (MSCs) could be good candidates for the treatment of ED [9].

MSCs can be isolated by several parts of human body, including bone marrow (BM), adipose tissue (AT), Wharton’s Jelly (WJ) tissue, umbilical cord blood (UCB), and neonatal teeth [10,11,12]. They are known for their immunoregulatory and immunosuppressive functions and have been administrated in patients in various clinical conditions. In an effort to establish their regenerative properties, they have been used in tissue engineering and regenerative medicine approaches [10,11,12]. Several clinical studies have been published so far presenting encouraging results in the treatment of ED with stem cells [13,14,15,16,17,18,19,20].

Autologous PLP (Platelet Lysate Plasma) has been used in regenerative medicine with promising results. PLP contains several growth factors such as platelet derived growth factor (PDGF), vascular endothelial growth factor (VEGF), epidermal growth factor (EGF), insulin-like growth factor (IGF), transforming growth factor-11 (TGF-11) and platelet-derived angiogenesis factor (PDAF) which are derived from platelets [21,22]. Clinical efficacy of PLP depends on several effects induced by these growth factors that may induce wound healing and regeneration of damaged tissues. More specifically, the efficacy on ED has been studied but more research is needed. [23,24].

The beneficial effect of stem cells and PRP in the tissues and erectile function prompt us to combine these methods in order to measure their efficacy and their safety.

## 2. Materials and Methods

This study is a prospective phase 1, single center pilot study. All patients were informed of the study design and signed a written informed consent in accordance to Helsinki declaration. It has been approved by the Scientific Committee of Attikon Hospital (Ref No 006; Date of approval: 15 September 2016), Athens, Greece.

The study is part of a bigger study that includes three groups of ED patients (5 patients in each group) that have been treated with Stem Cells and PLP (Group A), stem cells only (Group B) and PLP only (Group C). In this paper we present the 6 months of results of Group A (stem cells + PLP).

### 2.1. Study Design and Participants

Inclusion criteria were organic ED due to diabetes mellitus, hypertension, hypercholesterolemia, and Peyronie disease. Exclusion criteria were psychogenic, neurologic or hormonal erectile dysfunction, injuries to penis other than Peyronie’s disease and all cases of cancer. All patients underwent hormonal evaluation (Testosterone, Estradiol, LH, FSH, PRL, FT3, FT4, TSH, α-FP, CEA, CA 19-9), metabolic evaluation (Glucose, Cholesterol, Triglycerides) and PSA. A CT scan of the abdomen, thorax and brain were performed in all patients in order to exclude other pathologies. ED was evaluated with penile triplex and through International Index of Erectile Function (IIEF-5) questionnaire. Triplex of the penis was performed with the use of an intracavernosal injection of 20 μg of the vasodilator alprostadil (PGE1) (Pfizer, New York, NY, USA) to cause vasodilation in the penile vasculature, thus resulting in erection. Peak Systolic Velocity (PSV) and End Diastolic Velocity (EDV) were measured every 5 min for a total period of 20 min. PSV and EDV both were measured in cm/s. The IIEF-5 questionnaire is a 5-item questionnaire used for the evaluation of erectile dysfunction [25]. According to this, ED was classified into five severity levels, ranging from none (22–25) through severe (5–7). In all patients, physical evaluation was performed in all follow-up visits and an andrological history regarding the overall sexual status, including morning erections etc. was obtained. It is used in most ED studies and it has recently been re-evaluated and translated once more [26].

Patients were evaluated before stem cell and PLP injection and at 4 times points after injection, that is, at the 1st, 3rd, 6th and 12th month. In each visit, blood samples were collected, triplex of the penis was performed and the IIEF-5 questionnaire was completed. CT scans were performed before injection and at the 12^th^ month visit in order to exclude other pathologies or possible changes from baseline. Patients’ characteristics are presented in Table 1.

### 2.2. Procedure

Stem cells were obtained from the fat of the abdominal wall and therefore were Adipose-Derived Stem Cells (ADSCs). PLP was prepared from blood samples taken from a peripheral vein in the arm.

ADSCs were reconstituted with 2 mL of PLP and transferred into 1.8 mL of sterile cryotube until infusion was performed. ADSCs plus PLP were infused to the penis with the base of penis clumped for a period of 10 min.

### 2.3. Isolation and Expansion of ADSCs

ADSCs were initially isolated from patients’ lipoaspiration samples, that were enrolled in this study. Isolation and expansion of ADSCs was performed in compliance with the Good Manufacturing Practice (GMPs) rooms provided by the Hellenic Cord Blood Bank (HCBB) of Biomedical Research Foundation Academy of Athens (BRFAA). Briefly, the lipoaspiration samples were incubated in an orbital shaker at 37 °C and 345 rpm for 4 h along with collagenase 1mg/ mL (Sigma-Aldrich, Darmstadt, Germany). After incubation, the collagenase was inactivated with Phosphate Buffer Saline (PBS 1 ×, Sigma-Aldrich, Darmstadt, Germany) and centrifuged at 500 g for 6 min. The supernatant was discarded and the cell pellet was reconstituted with complete medium (α-ΜΕΜ supplemented with 15% FBS, 1% P-S and 1% L-glutamine, Sigma-Aldrich, Darmstadt, Germany). The cell pellet was transferred to 75 cm^2^ tissue culture flask (Costar, Corning Life, Canton, MA, USA) and incubated at 37 °C and 5% CO_2_ until confluence was observed. Then, the cells were detached with 0.25% trypsin-EDTA solution (Sigma-Aldrich, Darmstadt, Germany), washed with PBS 1 ×, and replated to 175 cm^2^ flasks (Costar, Corning Life, Canton, MA, USA). The same procedure was repeated until the cells reached passage 3. The number of ADSCs was counted in an automated Countess (Invitrogen, California, CA, USA) using Trypan blue (Invitrogen, California, CA, USA).

### 2.4. Quality Control of ADSCs

Flow cytometric analysis of ADSCs was performed according to the following panel of monoclonal antibodies. Specifically, CD90, CD105, CD73, CD29, CD19, CD31, CD45, CD34, CD14, CD3, CD19, HLA-DR and HLA-ABC were used. The CD90, HLA-ABC, CD29, CD19, CD31, and CD45 were conjugated with fluorescein isothiocyanate (FITC), while CD105, CD73, CD44, CD3, CD34, CD14 and HLA-DR were conjugated with phycoerythrin. The monoclonal antibodies were purchased from Immunotech (Immunotech, Beckman Coulter, Marseille, France). The immunophenotypic analysis was performed in a Cytomics FC 500 flow cytometer coupled with CXP Analysis software (Beckman Coulter, Marseille, France).

In addition, trilineage differentiation towards “osteocytes”, “adipocytes” and “chondrocytes” was performed. Specifically, ADSCs were differentiated to “osteocytes”, “adipocytes” and “chondrocytes” using the StemPro Osteogenic (Cat. No A1007201, Gibco, ThermoFischer, Waltham, MA, USA), Adipogenic (Ca. No. A1007001, Gibco, ThermoFischer, Waltham, Massachusetts, MA, USA) and Chondrogenic (Cat. No A1007101, Gibco, ThermoFischer, Waltham, Massachusetts, MA, USA) differentiation kits, respectively. The ADSC differentiation was evaluated using Alizarin Red S (for calcium depositions, Sigma–Aldrich, Darmstadt, Germany), Oil Red O (for lipid droplets, Sigma-Aldrich, Darmstadt, Germany) and Alcian blue (for proteoglycans, Sigma-Aldrich, Darmstadt, Germany) stains, according to manufacturer’s instructions.

### 2.5. PLP Preparation

Peripheral blood used for the production of PLP was collected in CPDA vacutained tubes. Platelet Lysate Plasma (PLP) was prepared from peripheral whole blood after centrifugation at 160 g for 20 min at 20 °C. The supernatant was centrifuged at 420 g for 15 min at 20 °C and ¾ of the plasma volume was discarded, allowing only the platelet rich plasma. The PLP was stored at −80 °C until further use.

## 3. Results

**Figure 1:**
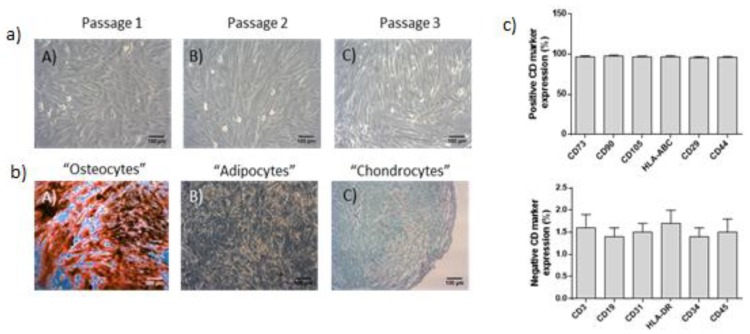
Expansion and quality control analysis of Adipose-Derived Stem Cells (ADSCs). (**a**) Morphology of ADSCs at passage 1 (A), 2 (B) and 3 (C). (**b**) Successful differentiation of ADSCs into “osteocytes” (A), “adipocytes” (B) and “chondrocytes” (C), as it was confirmed by Alizarin Red S, Oil Red-O and Alcian Blue, respectively. (**c**) Flow cytometric analysis of ADSCs, showing the positive and negative expression of specific CD markers. Images were obtained with original magnification 10 × and scale bars 100 μm.

ADSCs were successfully isolated from lipoaspirate samples from all patients. ADSCs presented spindle shaped morphology upon reached passage 3 (Figure 1). In addition, ADSCs positively expressed (>95%) CD73, CD90, CD105, HLA-ABC, CD29 and CD44, while negatively expressed (<3%) CD3, CD19, CD31, HLA-DR, CD34 and CD45 (Figure 1). ADSCs were successfully differentiated into “osteocytes”, “adipocytes” and “chondrocytes”. Specifically, calcium deposits produced from differentiated “osteocytes” were stained by Alizarin Red–S stain. Accordingly, lipid droplets and proteoglycans produced either from “adipocytes” or “chondrocytes” were stained by Oil-Red O and Alcian blue, respectively (Figure 1).

The number of stem cells injected is shown on Table 2. There is a difference in the number of the cells used between the first and the other patients. The reason is that in first patient the abdominal fat was collected with a punch biopsy but the number of expanded cells was not high enough and it was decided to perform a liposuction under anesthesia to the rest of the patients.

International Index of Erectile Function (IIEF-5) scores improved in all patients but patient 3. The pattern of improvement, although constant in these patients, is not similar to all. In patients 1, 2 and 5, there was a continuous improvement while in patient 4 there was a drop at 6th month (Table 3).

In all patients at the 6th month follow-up, there was an improvement at PSV but still the pattern was not similar to all patients during the 6-month period. Patient 1 and 2 had a decrease in the 1st month and then a continuous improvement, patient 3 and 5 had a continuous improvement from the first month and patient 4 had a continuous improvement with a decrease in the 6th month. Nevertheless, the 6th month results were better than baseline in all patients. On the contrary, EDV had a more variable pattern (Table 4).

Erectile function as perceived by the patients has improved over time as shown in Table 5. All the patients needed ICI before treatment in order to have an erection with the exemption of patient 2 who had no erections at all. During follow-up, there was an improvement that peaked on the 3rd month and remained stable till the 6th month. Patient 3 had a decreased in sexual function as there was a worsening in the erectile function between 3rd and 6th month.

There were no side effects noted in any patients during administration of stem cells or during the follow-up period except from a minor pain on the penis during injections that resolved on its own a couple of minutes later without additional treatment.

## 4. Discussion

Erectile dysfunction affects a large number of men and the current treatment cannot be considered curative as long as it is needed to take medication each time one needs to have sex. Regenerative therapy seems a promising treatment for many clinical conditions and clinical studies on the use of stem cells in the field of erectile dysfunction have made their appearance.

Stem cells by definition are cells that can renew themselves indefinitely but also give birth to other type of cells. There are several ways to characterize and classify stem cells. Based on their differentiation potential they are characterized as totipotent, which can give birth to all cell lines, pluripotent, which can differentiate to all germ layers but not to extra-embryonic cell lines, multipotent, which differentiate to all cell types within their germ layer and unipotent, which can differentiate to a specific cell line [27]. Based on their origin, they can be either Embryonic stem cells (ESCs) originating from embryonic tissue (inner cell mass of blastocyst) or Adult Stem Cells (ASCs) arising from adult tissue. ESCs are pluripotent while in general ASCs are considered multipotent. Recently though, it has been shown that ASCs can also be pluripotent [28]. Initially, bone marrow stem cells (BMSCs) were used, but the discovery of adipose derived stem cells (ADSCs) has replaced the BMSCs due to the simplicity of collection and the similar characteristics [29].

The first clinical study was published in 2010 from Korea and included seven diabetes patients that were proven unresponsive to previous medical therapies (oral phosphodiesterase 5 inhibitors, or PGE1 injection) for more than 6 months and all were awaiting penile prostheses. They were treated with human umbilical cord blood stem cells. The results were very encouraging—three patients experienced morning erections, 1 month after treatment and all but 1 regained morning erections by the second month; this was maintained for at least 3 months. Six patients experienced an increase in penile hardness and two could achieve penetration with the use of PDE-5I [13]. Several other studies have been published with promising results and ED improvement was noted in all these studies [14,15,16,17,18,19,20].

There are several questions that need to be addressed regarding the use of stem cells on treating erectile dysfunction. Firstly, which is the best “stem cell” that we can use? ADSCs have gained ground due to their abundance and their simplicity to collect them. However, in the published studies, several types (umbilical, placental, bone marrow, adipose-derived) have been used with similar results and without being able to support one over the other [13,14,15,16,17,18,19,20]. Secondly, should we use stem cells from the same patient or can we choose a more healthy donor? The first published study on ED used cells from a donor taken from a company that provided cells [13]. There are data that indicate that stem cells from a patient might not be as healthy as we would like them to be [30]. Although allogeneic stem cells have been used in some of the studies of ED, the regulations in most of the countries make it impossible at the moment. Third, should we used expanded stem cells in order to have big numbers or should we prefer the SVF (Stroma Vascular Fraction) that represents an early stage in the production of stem cells and includes other types of cells (pericytes, macrophages, endothelial cells, fibroblasts, endothelial precursor cells) which make it possibly more effective [31]? The majority of the studies on ED use SVF and we believe this is because it is easier to produce SVF as it does not need culture and expansion.

As long as there is no proof that one type of stem cell is better than the other, adipose stem cells have the advantage of easiness on collection through liposuction of the adipose tissue from the abdominal wall. This is the reason that this type was used in our study. Another concern is the use of SVF or expansion of cells through culture for the reasons earlier described. In our study we used a method that combines the advantages of SVF and expanded stem cells. We cultured the cells in order to obtain big numbers of ADSCs but we also used PLP which is high in growth factor in an effort to mimic the SVF ingredients. PRP seems promising in treating ED and we decided to combine both in order to increase efficacy as it is feasible without negative reactions between these two [31]. This is the only study so far that uses this method as a possible regenerative treatment for ED. The results are promising. The IIEF-5 score has increased in all but one patient (patient 3). This patient, although he considered that his erections improved in the pro-stem cell period, still had a low IIEF-5 score. This was initially attributed to the fact that questions on the IIEF-5 questionnaire refer to the “erections during intercourse”. This patient due to personal reasons did not have sex and so he responded negatively, providing a low score. But during the 6th month follow up, this patient noticed deterioration in his erections, meaning that stem cell treatment was not so effective for him. Peak systolic velocity improved in all patients, reflecting an improvement in the endothelial function of the penis. This was obvious to the erectile function, as the patients before treatment needed ICI in order to obtain an erection or they had no erections at all (Patient 2) while after treatment they were able to have erections either on their own or with the use of tablets (PDE5-I) instead of ICI. Although unassisted erections would be the ideal for a man, being able to have erections with a tablet instead of an injection is a significant improvement as well.

Our results are in accordance with the other published studies [13,14,15,16,17,18,19,20], and although there are differences in the cells used, it seems that stem cell treatment has till now positive results in erectile function. It is also very important that patients treated in the published studies had different causes of ED including mainly diabetes and radical prostatectomy or other not-specified causes and the results are in all cases positive although not similar in improvement. This is very encouraging because it shows that regenerative therapy has a beneficial effect in many causes of ED.

The possible effect of low-intensity shock wave treatment has been recently presented [32]. Therefore, this offers a possible additional benefit if stem cell treatment is combined with shock wave sessions but more studies are needed in the field.

The durability of this treatment is so far in accordance to the other studies. After 6 months of stem cell administration, the improvement in erectile function remained in all but one patient. That means that with one treatment a man can have an improvement in his erections lasting for half a year. Although further follow-up is awaited, this is a significant result so far. The other studies provide similar results with ED improvement lasting more than 6 months, but not in all the patients. Therefore, it seems that stem cell therapy is not a radical therapy but nevertheless offers a remarkable durability. The question of readministration has been addressed in one study so far [31] with similar efficacy without any side effects.

Of great importance is the safety profile of the treatment. Till the 6th month period, there were no side effects noted apart from a minor pain in the penis which resolved on its own. This is in accordance with the other studies on the use of stem cells for ED that also reported no side effects.

There are some limitations in our study. Although innovative in its design, it includes few patients. Thus, at this stage we cannot predict who will benefit from such a treatment although it seems than it can be offered to most of the patients (taking in considerations our exclusion criteria). Another interesting observation is patient 1, who although having less stem cells than the other patients, had similar positive results. This can be attributed to the presence of PLP and its growth factors or simply this patient might have been even better with more cells. Furthermore, a significant limitation is the absence of control group. Due to the invasive nature of the treatment, it is not easy to include such a group and therefore we cannot rule out the placebo effect. However the results in our study are positive and they are in accordance with the other studies in the field.

## 5. Conclusions

Stem cell therapy in combination with PLP appears to show some improvement in erectile function and has minimal side effects in the short term. The results are very promising but it is early to generalize this treatment and it should be used in controlled settings only by experts on the field.

## Figures and Tables

**Table 1:** Patients’ characteristics.

Patient	Age	Comorbidities
Patient 1	62	Hypertension, Hypercholesterolemia
Patient 2	52	Diabetes, Hypercholesterolemia
Patient 3	52	Hypertension, Diabetes
Patient 4	66	Peyronie’s disease
Patient 5	52	Diabetes, Hypertension, Hypercholesterolemia

**Table 2:** Number of stem cells injected. (× 10^6^).

Patient	Stem Cells Injected
1	9.5
2	43.2
3	37.2
4	53.2
5	51.4

**Table 3:** IIEF-5 scores.

Patient	Before Injection	1st Month	3rd Month	6th Month
1	6	17	12	23
2	10	12	12	15
3	6	5	6	8
4	14	20	22	21
5	16	16	20	22

**Table 4:** Triplex results, presented as (PSV)/(EDV): Peak Systolic Velocity (PSV)/End Diastolic Velocity (EDV), measured at cm/sec.

Patient	Before Injection	1st Month	3rd Month	6th Month
1	35/11	30.5/7.8	39/12	42/10.5
2	40.3/8.6	25.4/ 6.0	40.8/11.0	59.4/ 14.4
3	16.1/4.7	35/8.9	61.2/20.6	65.2/14.9
4	57/15	78.2/16.6	97.9/22	62.8/13.8
5	45.5/17.8	49.4/14.7	62.6/25.8	83/35

**Table 5:** Erectile function.

Patient	Erectile Function before Therapy	Erectile Function 1st Month	Erectile Function 3rd Month	Erectile Function 6th Month
1	Only with ICI, unable to climax	Hard erections with oral PDE5-I, normal ejaculation	Hard erections with oral PDE5-I, normal ejaculation	Hard erections with oral PDE5-I, normal ejaculation
2	No erections	Some increase in hardness	Hard erections with oral PDE5-I	Hard erections with oral PDE5-I
3	Moderate erections with ICI	Improvement only in morning erections	Hard erections with oral PDE5-I	Decreased on the hardness of erections with oral PDE5-I
4	Moderate erections with ICI	Hard erections with oral PDE5-I	Unassisted hard erections	Unassisted hard erections
5	Hard erections only with ICI	Unassisted hard erections short duration	Unassisted hard erections	Unassisted hard erections
